# Case report: Rapidly progressive desmoid tumor after surgery for esophagogastric junction cancer and slowly progressive primary desmoid tumor: a report of two cases and literature review

**DOI:** 10.3389/fonc.2024.1401839

**Published:** 2024-05-10

**Authors:** Chuan-Ying Li, Yan-Peng Gao, Ming-Hao Jia, Yuan Zhou, Jia-You Xu

**Affiliations:** ^1^ School of Clinical Medicine, Shandong Second Medical University, Weifang, Shandong, China; ^2^ Department of Gastrointestinal Surgery, Weifang People’s Hospital, Weifang, Shandong, China

**Keywords:** desmoid tumor, soft tissue tumors, surgical treatment, mesentery, case report

## Abstract

**Background:**

Desmoid tumor (DT) is a rare locally aggressive but non-metastatic mesenchymal soft tissue neoplasm that predominantly occurs in the abdominal wall, abdominal cavity, and extremities. Its occurrence in the mesentery is relatively uncommon.

**Case reports:**

This article reports two cases of desmoid tumor treated at the Department of Gastrointestinal Surgery, Weifang People’s Hospital. The first case was a 59-year-old male patient who had previously undergone surgery for esophagogastric junction cancer. Postoperatively, he developed an intra-abdominal mass that rapidly increased in size within three months. The second case was a 60-year-old male patient who incidentally discovered a mass in the left lower abdomen. Both patients underwent surgical treatment, and the postoperative pathological diagnosis was mesenteric desmoid tumor.

**Conclusion:**

The treatment of desmoid tumor remains challenging. Simple surgical resection often yields unsatisfactory outcomes, and the efficacy of adjuvant radiotherapy and chemotherapy is also limited. Further research and clinical practice are necessary to improve diagnostic and therapeutic strategies, aiming to enhance patient survival and quality of life.

## 1 Introduction

Desmoid tumor, also known as aggressive fibromatosis, is a rare locally aggressive but non-metastatic soft tissue neoplasm. The annual incidence is 2-4 cases per million population, accounting for approximately 0.03% of all neoplasms and less than 3% of all soft tissue tumors (1). Mesenteric desmoid tumors are relatively rare and often difficult to diagnose due to the lack of specific clinical symptoms, leading to frequent misdiagnosis. Here, we report a highly unusual case of mesenteric desmoid tumor following surgery for esophagogastric junction cancer. This case is extremely rare and easily misdiagnosed as tumor recurrence and metastasis. The rapid growth observed in this clinical scenario contributes to a deeper understanding of the infiltrative nature and progression patterns of this disease. Furthermore, a comparative analysis with a common case of mesenteric desmoid tumor is presented, elucidating the clinical characteristics and management strategies for patients with different clinical presentations of desmoid tumor. A review of the literature on the current diagnosis and management of this condition is also provided.

## 2 Case presentation

### 2.1 Case 1

A 59-year-old male patient was diagnosed with Siewert type III esophagogastric junction cancer on November 22, 2021. Pathological examination revealed moderately differentiated adenocarcinoma. Immunohistochemistry showed Programmed Death-Ligand 1 (PD-L1) [22C3 assay, Combined Positive Score (CPS) = 5] positivity. Based on imaging investigations, the initial clinical staging identified the disease as T4aN3aM0. Subsequently, the patient underwent 4 cycles of neoadjuvant therapy with CAPOX (capecitabine + oxaliplatin) + PD-1 (programmed cell death protein 1, sintilimab). On February 25, 2022, he underwent total gastrectomy with Roux-en-Y esophagojejunostomy at Beijing Cancer Hospital. Postoperative pathology revealed pathologic complete response (ypT0N0M0) of the previously diagnosed moderately differentiated adenocarcinoma. He had an uneventful postoperative recovery and did not receive adjuvant therapy. He remained disease-free during the 1-year follow-up.

**Figure 1:**
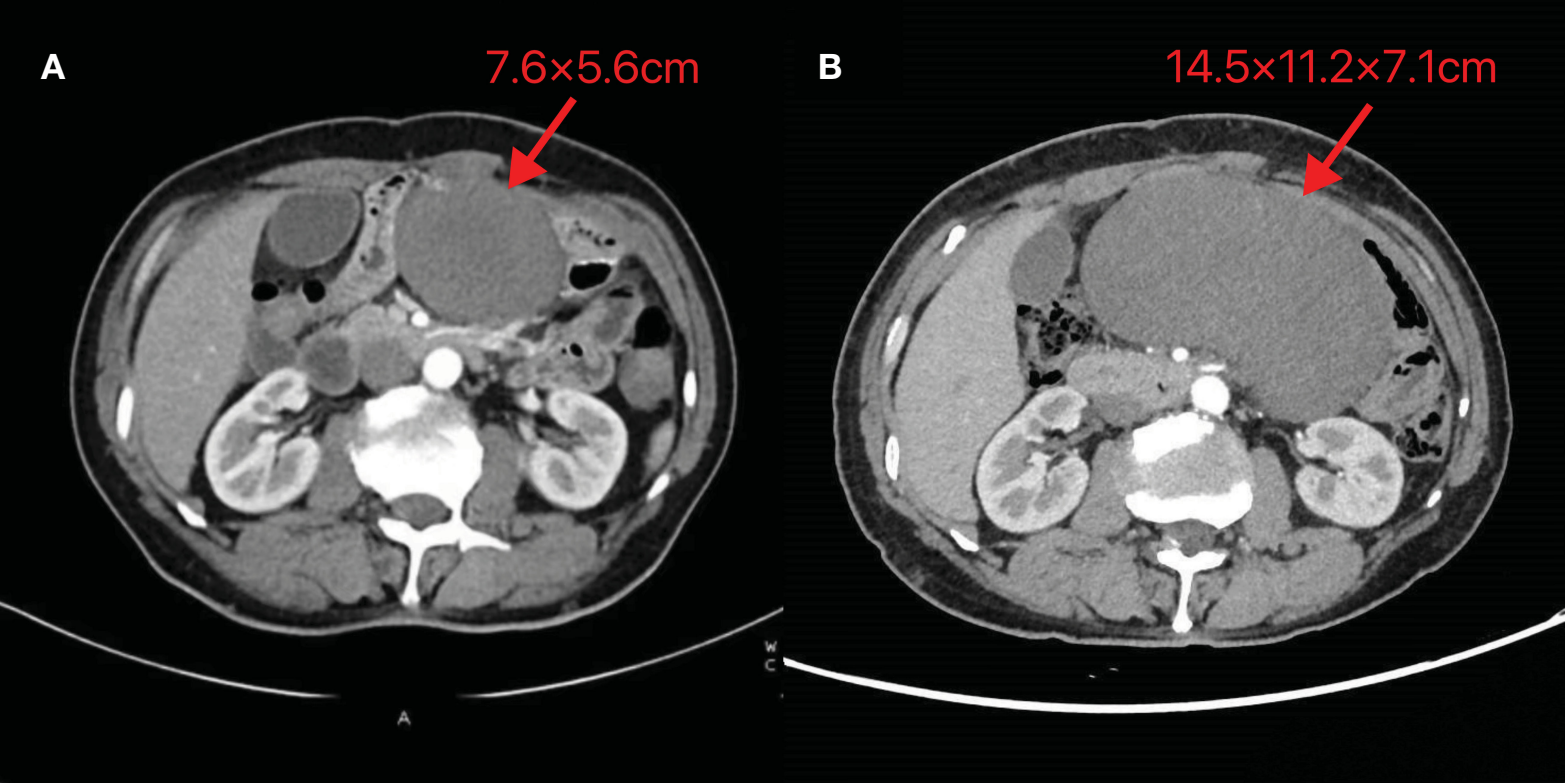
Abdominal CT images of Case 1 **(A)** Taken on 2023-10-24 **(B)** Taken on 2024-01-15. The image reveals a mass-like low-density shadow in the left abdomen with well-defined borders, surrounded by vascular structures at the edges. The maximum cross-section recorded on 2023-10-24 is 7.6×5.6cm, while the maximum size observed on 2024-01-15 is 14.5×11.2×7.1cm.

On October 24, 2023, routine follow-up abdominal contrast-enhanced CT revealed a left abdominal mass (7.6×5.6cm), initially diagnosed as metastatic lymph node involvement (**Figure 1A**). Percutaneous biopsy demonstrated a spindle cell proliferative lesion. Immunohistochemistry showed CD117 (–), CD34(+in vessels), CK (-), Desmin (-), DOG-1(-), Ki67(+5%), S-100(-), SMA (-), and STAT6(-). When initially diagnosed with a potential tumor recurrence, the patient experienced profound anxiety and panic. However, upon histopathological examination ruling out this diagnosis, he felt a sense of relief. As there was no discomfort, the patient opted for observation. On January 15, 2024, he was admitted due to a mild pain in the left abdomen. Laboratory tests were normal, and abdominal CT showed a left midabdominal low-density mass (14.5×11.2×7.1cm), diagnosed as a mesenteric smooth muscle tumor (**Figure 1B**).

**Figure 2:**
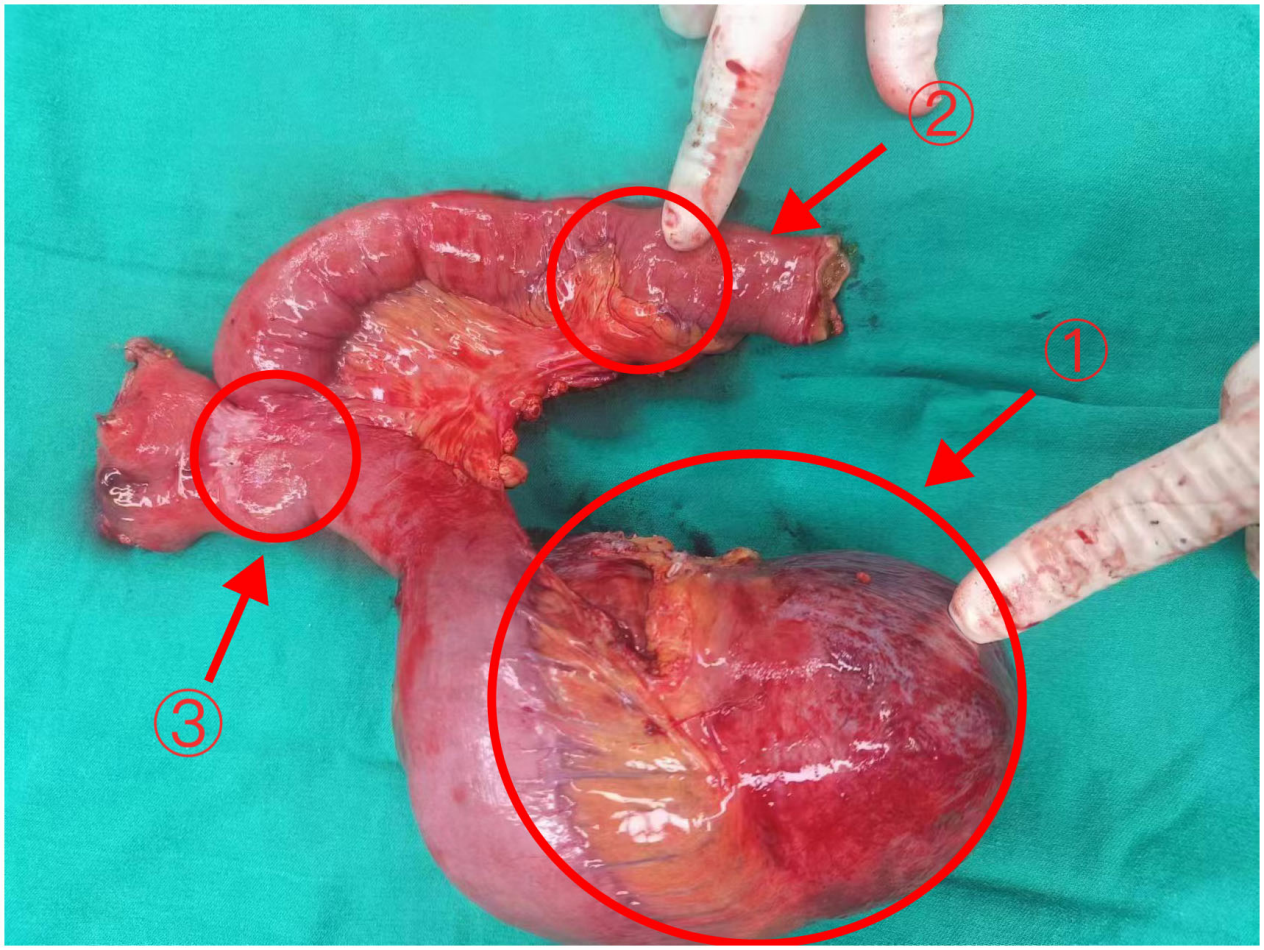
The tumor and part of the jejunum of Case 1 Arrows 1: The larger tumor (18×12×9cm). Arrows 2: The smaller tumor (3.5×3×2cm) Arrows 3: The original Braun anastomosis.

On January 17, 2024, an exploratory laparotomy was performed to address the tumor’s rapid growth and prevent further enlargement and potential harm to intra-abdominal organs. Intraoperatively, the tumor was located in the mesentery distal to the Braun enteroenterostomy, measuring approximately 15×12cm, with a firm and friable texture, closely related to the mesenteric vessels of the efferent loop and afferent loop. Another 4×3cm mass was noted in the mesentery of the efferent loop, 15cm distal to the Braun anastomosis. The procedure involved careful dissection of the tumor from surrounding adhesions, sequential ligation of the tumor’s feeding vessels, transection of the jejunum 10cm from the ligament of Treitz, and transection of the jejunum 20cm distal to the Braun anastomosis using a stapling device. Completely cut off the tumor and part of the jejunum (including the original Braun anastomosis) (**Figure 2**). The esophagus was transected 1cm proximal to the previous esophagojejunostomy. An end-to-side esophagojejunostomy was performed using a circular stapler, and the afferent loop was closed with a stapling device. A side-to-side jejunojejunostomy was created between the afferent loop and efferent loop, approximately 40cm distal to the esophagojejunostomy, and the common opening was closed with sutures.

**Figure 3:**
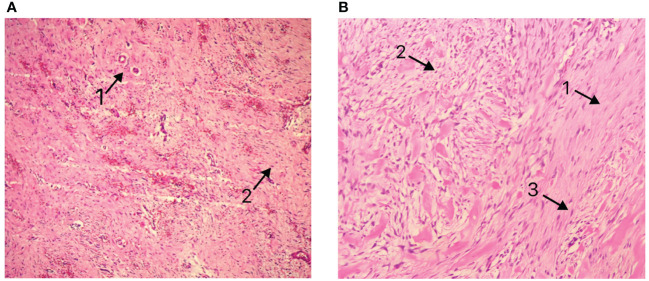
**(A)** Pathological presentation of Case 1 **(B)** Pathological presentation of Case 2. Arrows 1: Matrix composed of abundant interwoven collagen fibers. Arrows 2: Spindle/ovoid-shaped fibroblast cells. Arrows 3: Elongated cell nuclei without significant atypia.

Postoperative pathology revealed a mesenchymal tumor consistent with a desmoid tumor, with two lesions: a larger tumor (18×12×9cm) invading the surrounding adipose tissue and a smaller tumor (3.5×3×2cm) invading the muscularis propria of the bowel wall and surrounding adipose tissue, without mucosal involvement, perineural invasion, or vascular tumor thrombus. Resection margins were negative (**Figure 3A**). Immunohistochemistry of the larger mass showed Vimentin (+), CD117(-), DOG-1(-), CD34(+in vessels), SMA (focally+), Desmin (-), SOX10(-), S-100(-), β-catenin (nuclear+in some cells), ALK (-), and Ki-67 index of 1%. The smaller mass showed β-catenin (nuclear+in some cells).

**Figure 4:**
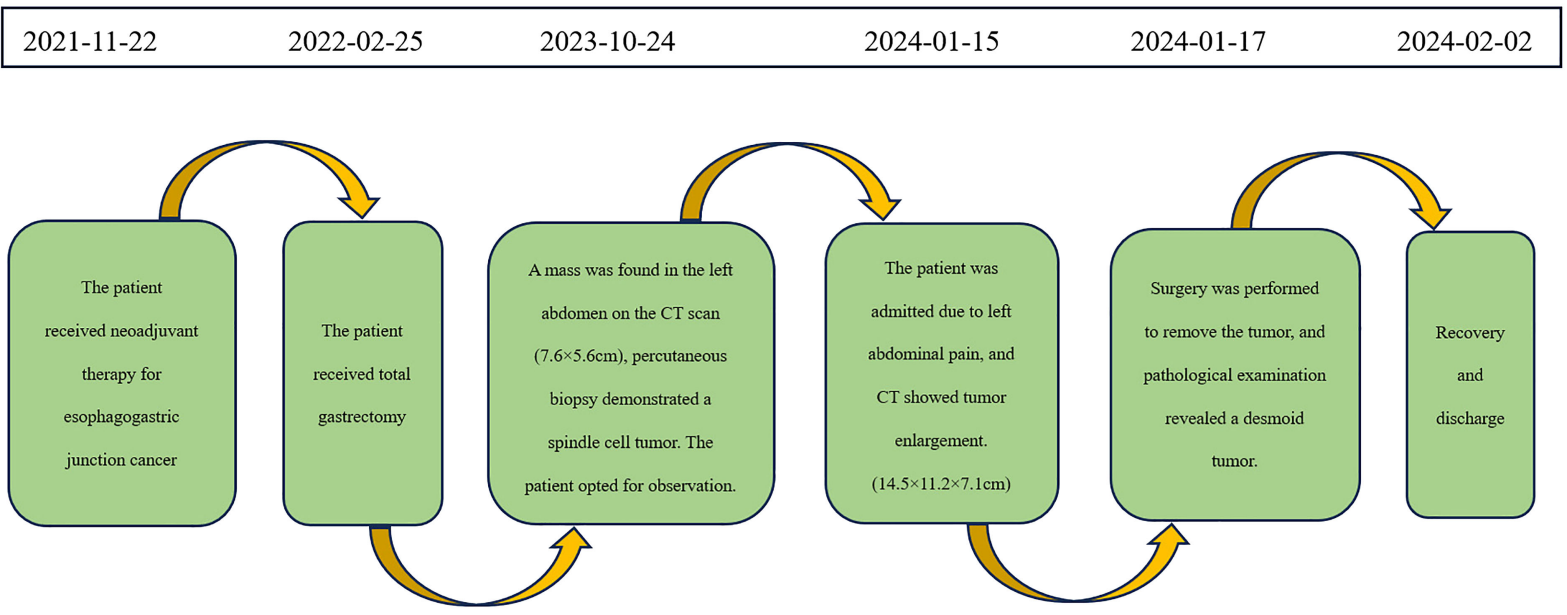
Timeline for Case 1.

The patient had an uneventful recovery and was discharged on February 2, 2024. The timeline of relevant diagnoses and treatments is showcased in **Figure 4**.

### 2.2 Case 2

**Figure 5:**
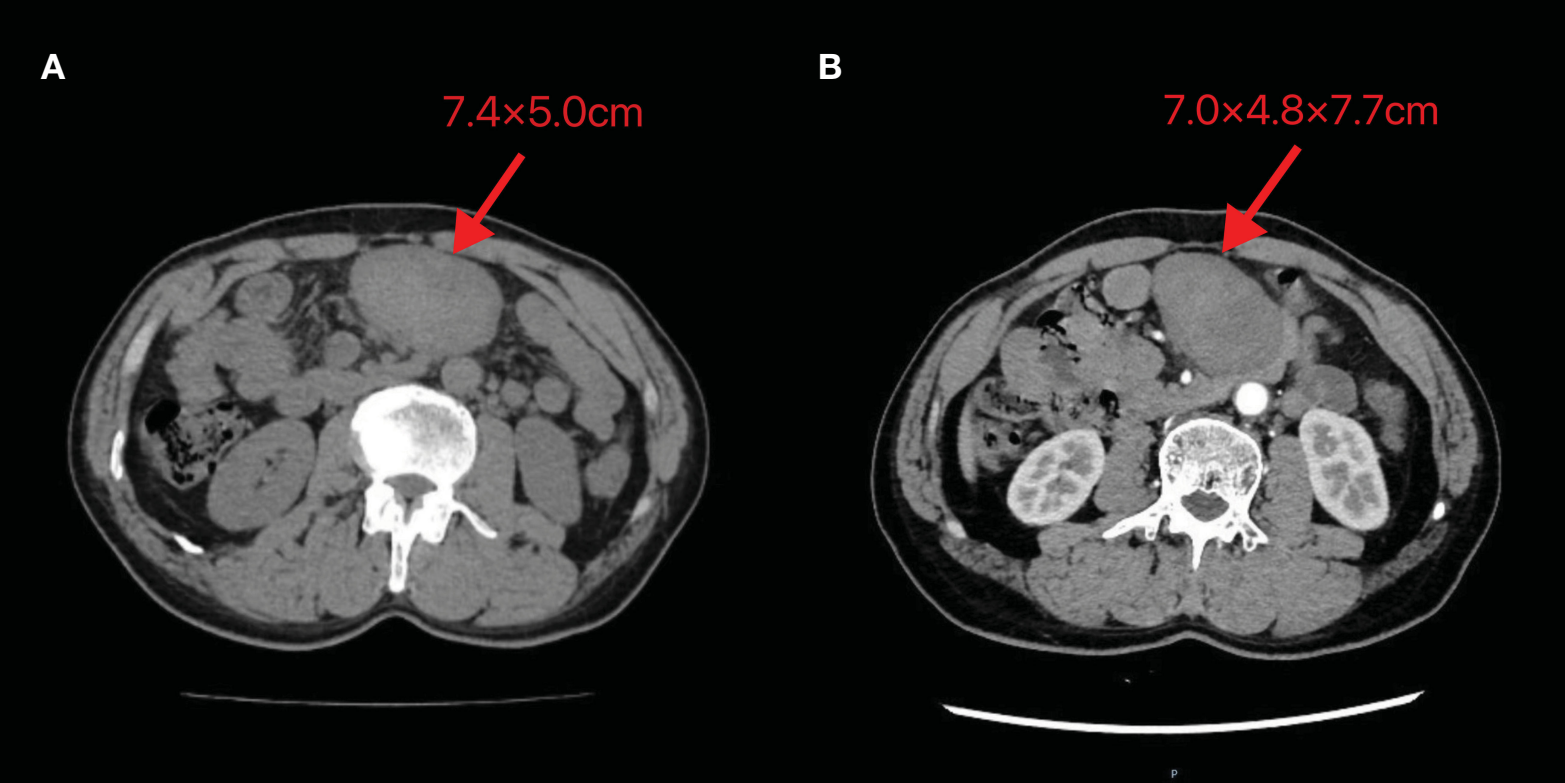
Abdominal CT images of Case 2 **(A)** Taken on 2024-01-05 **(B)** Taken on 2024-02-20. The image shows a mass of soft tissue density behind the transverse colon in the upper abdomen, with uneven internal density and unclear demarcation from the adjacent small intestine. The maximum cross-section recorded on 2024-01-05 is 7.4×5.0cm, while the maximum size observed on 2024-02-20 is 7.0×4.8×7.7cm.

A 60-year-old male patient incidentally touched a mass in the left lower abdomen without any discomfort. On January 5, 2024, an abdominal CT scan revealed a space-occupying lesion (7.4×5.0 cm) in the left lower abdomen (**Figure 5A**). As there was no discomfort and the CT scan did not show the specific nature of the mass, the patient was followed up for observation and did not receive any specific treatment. On February 20, 2024, despite having no symptoms, the patient decided to be admitted for surgical treatment to prevent potential progression after careful consideration. A repeat abdominal enhanced CT scan demonstrated a soft tissue density lesion (7.0×4.8×7.7 cm) in the mesenteric region of the lower abdomen (**Figure 5B**).

As malignancy could not be ruled out, and there were concerns that further growth could lead to life-threatening complications, the patient decided to undergo upfront surgery rather than a biopsy. On February 23, 2024, an exploratory laparotomy was performed. Intraoperatively, a tumor of approximately 10×8 cm was identified in the retroperitoneal region of the left lower abdomen. The tumor had an intact capsule, a firm consistency, and a solid nature. It was located approximately 5 cm from the start of the jejunum and was closely connected to the jejunum and its mesenteric vessels. The surgical procedure involved dissecting the adhesions between the small bowel mesentery and the tumor, separating along the tumor capsule layer, and ligating the blood vessels supplying the tumor. The ligament of Treitz was dissected and the jejunum was transected at its origin. An anvil of a circular stapler is inserted into the proximal jejunal stump. The jejunum was also transected at a distance of 10cm from the tumor, and an end-to-side jejunojejunostomy was performed using the circular stapler. The residual ends were closed.

**Figure 6:**
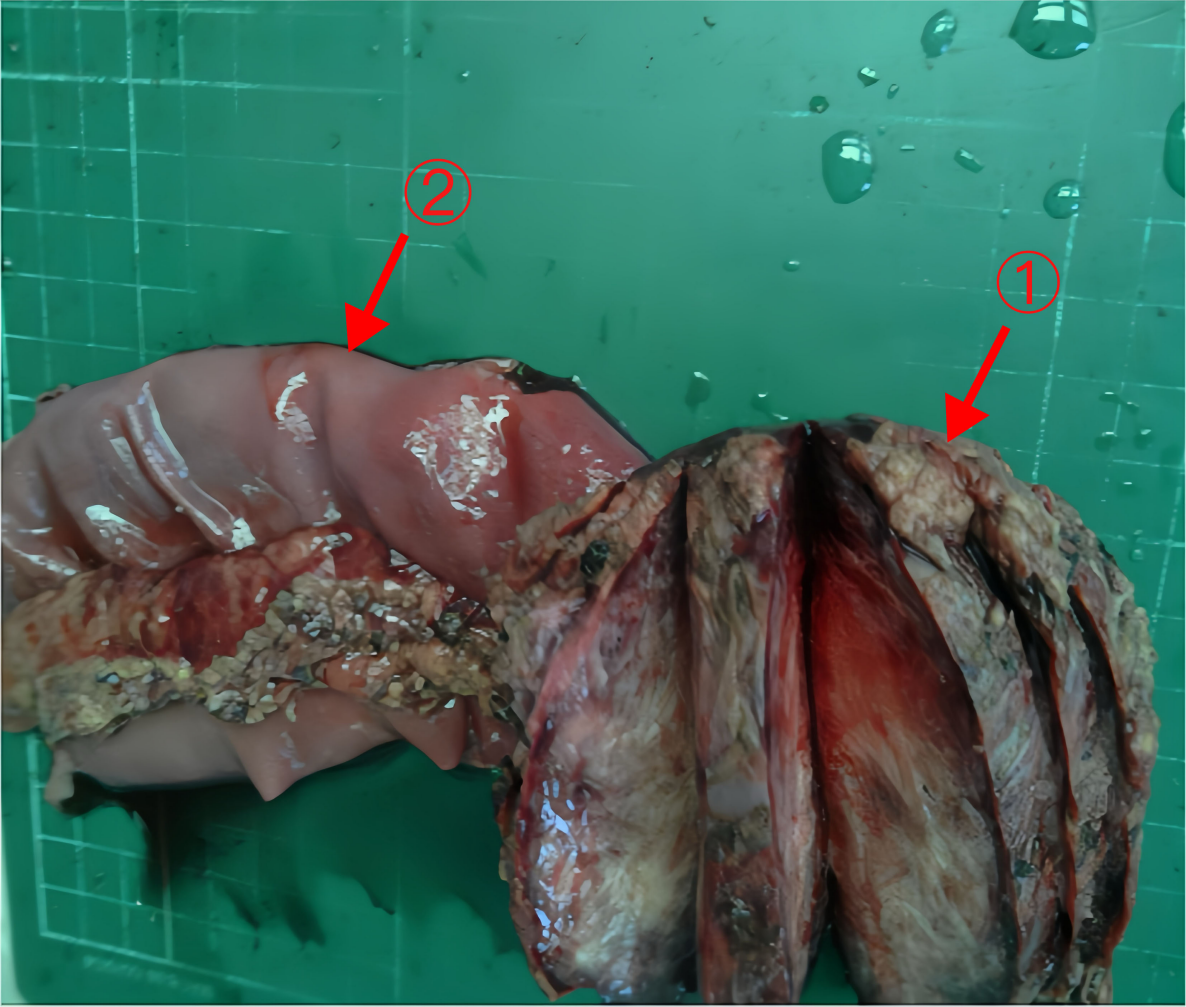
The tumor and part of the jejunum of Case 2 Arrows 1: The tumor (8.5×7×5cm). Arrows 2: Part of the jejunum (20cm).

The postoperative pathology report revealed a spindle cell lesion within the muscular layer of the bowel wall, accompanied by mucinous degeneration, consistent with a desmoid tumor. The tumor measured 8.5×7×5 cm (**Figure 6**), and five peri-intestinal lymph nodes showed reactive hyperplasia. Both resection margins were negative (**Figure 3B**). Immunohistochemical findings were as follows: Vimentin (+), β-catenin (partial nuclear positivity), SMA (partially positive), Desmin (+), CD34 (vascular positivity), SDHB (scattered positivity), CK (broad) (-), STAT6 (-), S-100(-), CD117(-), DOG-1(-), ALK (-), Ki-67 index 5%.

**Figure 7:**
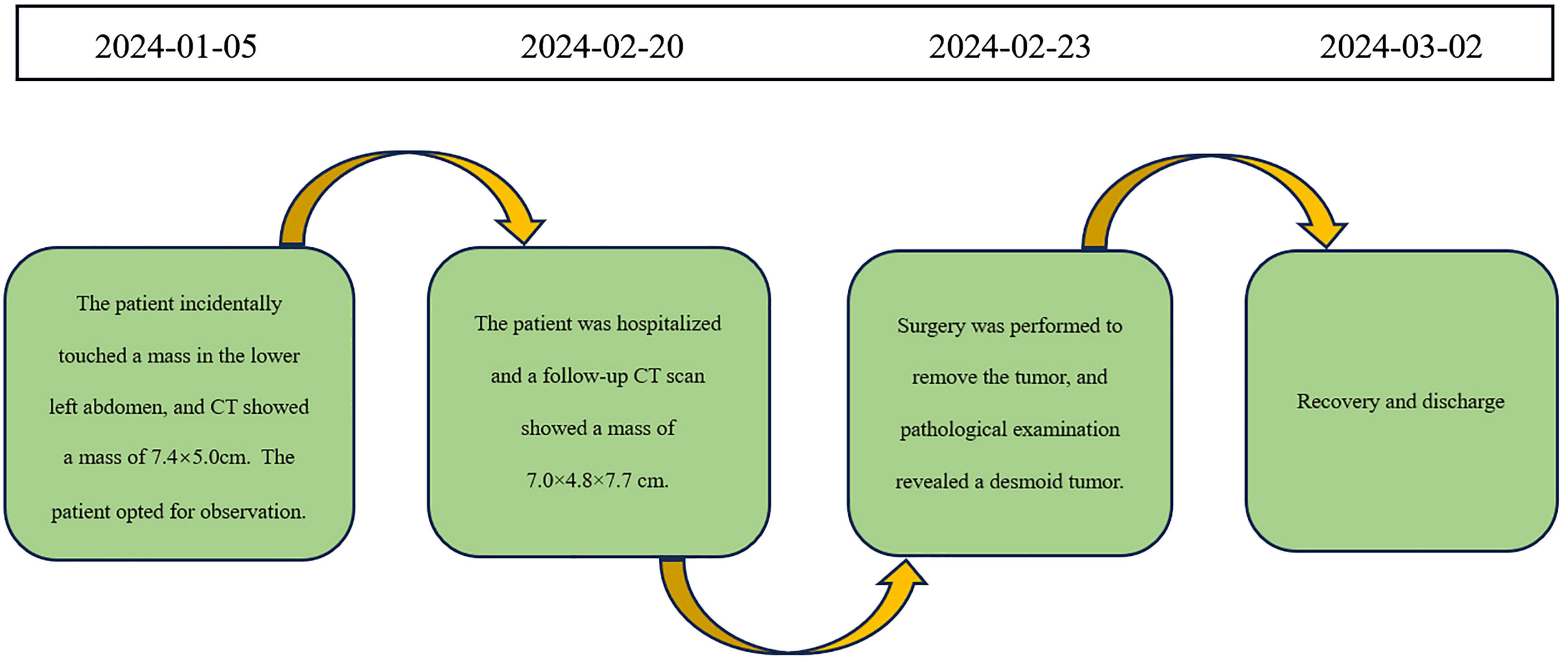
Timeline for Case 2.

The patient recovered well and was discharged on March 2, 2024. The timeline of relevant diagnoses and treatments is showcased in **Figure 7**.

## 3 Discussion

### 3.1 Epidemiology

Desmoid tumor originates from mesenchymal tissue and is a rare clonal proliferative neoplasm of fibroblastic cells. The World Health Organization (WHO) classifies it as a borderline tumor, characterized by aggressive local invasion, a high recurrence rate, and local destructive potential (2). This disease typically affects individuals in the age range of 30-40 years, with a higher incidence in females.

Desmoid tumor can occur in any part of the body and is classified into extra-abdominal, abdominal wall, and intra-abdominal types based on the site of origin, with the intra-abdominal type being the least common (3). The most frequent sites of involvement include the trunk, extremities, abdominal wall, and intra-abdominal cavity (commonly involving the mesentery or retroperitoneum). In contrast to the reported high-risk population, the two cases described in this article were elderly males, suggesting a broader susceptibility range for this disease. The lesions in both patients were located in the left upper abdomen, encasing the surrounding small bowel mesentery and adhering closely to the mesenteric vessels, representing a relatively uncommon form of mesenteric desmoid tumor.

### 3.2 Etiology and pathogenesis

In addition to the epidemiological features, the pathogenesis of desmoid tumors is also worth investigating. The exact pathogenic mechanism of desmoid tumor remains unclear, but it may be associated with factors such as abdominal surgery, trauma, estrogen levels, Gardner’s syndrome, and Crohn’s disease. Most cases of desmoid tumor are sporadic, with more than 85% of sporadic cases harboring CTNNB1 gene mutations. Approximately 5%-10% of cases are associated with familial adenomatous polyposis (FAP) syndrome, which is characterized by APC gene mutations (4).

Estrogen plays a role in the development and progression of desmoid tumor. Estrogen can stimulate tumor growth, and in most cases, tumor cells express estrogen receptor β, which may contribute to the higher incidence in females (5). Additionally, research suggests that open surgical trauma is a risk factor for this disease. Surgical injury can induce muscle fiber hemorrhage and hematoma formation, releasing inflammatory and growth factors that promote tumor development. In contrast, laparoscopic surgery has been shown to reduce the risk of developing desmoid tumor after prophylactic surgery in patients with FAP (6).

In the two cases reported in this article, both patients were male and had no family history of FAP. For Case 1, the patient had previously undergone open total gastrectomy, suggesting that surgical trauma-induced tissue damage may have been an important contributing factor. As for Patient 2, further investigation is needed to uncover the etiology, illustrating the complexity of the pathogenesis of desmoid tumors.

### 3.3 Clinical manifestation

Variations in the pathogenesis may result in diverse clinical presentations of desmoid tumors. Patients with desmoid tumors exhibit varying clinical manifestations depending on the location of the tumor growth. Lesions in the extremities may compress surrounding nerves, leading to numbness and impaired joint mobility. Intra-abdominal lesions often compress adjacent organs, causing symptoms such as abdominal distension, pain, intestinal obstruction, hematuria, or hematochezia, but these symptoms lack specificity. Pain is a common symptom of desmoid tumor and is often related to the size and location of the tumor. Studies have shown that one-third of confirmed desmoid tumor patients experience pain, particularly those with tumors larger than 5 cm or located in the neck or shoulder region (7). In these current cases, neither patient felt significant pain, probably because the affected area was non-invasively located in the mesentery. Mesenteric desmoid tumor often presents without obvious clinical symptoms, with a few cases presenting with incidentally detected masses, abdominal pain, fatigue, or gastrointestinal symptoms such as nausea.

In the two cases reported in this article, both patients incidentally discovered abdominal masses during physical examinations, which revealed mobile, non-pulsatile, and non-tender masses in the left upper abdomen. Case 1 had a history of curative surgery for esophagogastric junction cancer and developed an intra-abdominal mass 20 months later. Given the patient’s history of cancer surgery, our initial diagnosis was tumor recurrence or metastasis. However, the occurrence of desmoid tumor after curative gastrectomy for gastric cancer is an extremely rare situation, with few reported cases (8, 9). Therefore, a definitive diagnosis could only be made after the postoperative pathological results confirmed desmoid tumor.

The clinical course of desmoid tumor is often unpredictable, with possible spontaneous regression, temporary stabilization, or further progression. Although most cases exhibit relatively slow growth, exceptions with rapid expansion can occur. A case series study found that desmoid tumor typically grows gradually over a median period of 3 years before stabilizing, with recurrence or progression most commonly occurring between 14-17 month (10). The tumor in case 1 of this article doubled in volume within 3 months, and the rapid growth rate is a rare occurrence. This breaks the stereotype that desmoid tumors typically show slow growth. The tumor volume of patient 2 tends to stabilize, which is consistent with the slow progression described in the literature. This difference reminds us that there are indeed different biological behaviors in desmoid tumors and reminds us to be alert to the special situation of case 1 to avoid misdiagnosis.

### 3.4 Diagnosis

Early and accurate diagnosis of desmoid tumor is crucial, but it is often challenging due to the rarity of the disease and the lack of experience among clinicians and radiologists. This can lead to a high rate of misdiagnosis and delayed treatment. Ultrasonography, computed tomography (CT), and magnetic resonance imaging (MRI) are the main diagnostic tools. They help determine the location, size, extent, and local invasion of the lesion, providing a reference for surgical treatment planning (11).

Ultrasonography is safe, non-invasive, and cost-effective, making it a preferred initial screening method and an important tool for detecting recurrence after treatment. CT can reveal the surrounding tissue conditions but has a relatively low soft tissue resolution. MRI offers superior soft tissue resolution, clearly depicting the composition of the tumor and providing a high diagnostic accuracy. Additionally, MRI can evaluate the extent of tumor invasion and its relationship with adjacent structures, facilitating successful surgery. Pathological examination of the tumor tissue is essential, and core needle biopsy is recommended to obtain adequate tumor tissue for evaluation by experienced pathologists (12). In the case 1, preoperative ultrasound-guided percutaneous biopsy of the abdominal mass was performed, but the diagnosis was missed due to inadequate tissue sampling, resulting in a delay in appropriate treatment. In addition, the patient in case 2 directly chose surgical resection, regardless of whether the tumor was malignant or not, to prevent potential tumor progression, so no biopsy was performed. Histologically, desmoid tumor exhibits bundles or woven sheets of spindle cells with a collagen-rich stroma. Immunohistochemistry often shows positivity for vimentin and β-catenin, with frequent positive staining for smooth muscle actin (SMA) and desmin. Common tumor markers such as CD34, CD117, DOG-1, and S-100 are typically negative (13). The pathological and immunohistochemical findings in the present cases were consistent with the literature, and these features are essential for confirming the diagnosis and differentiating desmoid tumor from other types of tumors.

Overall, the diagnosis of desmoid tumors is challenging and the specificity of existing imaging and pathological techniques is limited. As described in this article, even with imaging and biopsy, the lack of specificity in their presentation may lead to initial misdiagnosis in both cases. This highlights the urgent need to develop more accurate imaging and molecular biological methods to improve the accuracy of diagnosis of desmoid tumors and provide a basis for formulating appropriate treatment plans.

### 3.5 Treatment

#### 3.5.1 Observation and surgery

Once diagnosed with a desmoid tumor, it is crucial to select an appropriate treatment method. Surgical resection was once considered the standard first-line treatment, but the current international consensus favors active monitoring as the primary treatment approach, followed by systemic therapy, unless the condition threatens life or vital organs (14, 15). In 2020, the Desmoid Tumor Working Group issued guidelines recommending active surveillance as the preferred initial approach for asymptomatic or mildly symptomatic, non-progressive, or non-life-threatening tumors. The guidelines suggest initiating observation after histological diagnosis and imaging evaluation, starting with an initial imaging scan followed by continuous monitoring within 1-2 months and subsequent follow-ups every 3-6 months. Active treatment is advised exclusively for patients exhibiting continuous progression and/or worsening symptoms. Surgical resection is recommended for cases in which tumor growth may lead to organ dysfunction (16–18). Xie et al. suggested that desmoid tumor originating from the small intestine is extremely rare, locally aggressive, and challenging to diagnose and treat. They recommended surgical resection followed by a “watch-and-wait” strategy with regular follow-up to prevent rapid tumor growth and subsequent damage to abdominal organs (19). The cases reported in this article are similar to the description by Xie et al. In Case 1, the tumor exhibited rapid growth, representing a continuous progression risk, so we performed surgical resection for him. This also aligns with the indications for surgical treatment summarized by Improta et al, including no diagnostic/diagnostic doubt and significant tumor size/life-threatening progression (20). However, in Case 2, although the tumor volume was initially stable, the patient opted for surgical resection without biopsy to prevent potential tumor progression that could threaten his life. Therefore, we also opted for surgical resection with periodic follow-up. At the same time, this case reminds us of the need to respect patients’ subjective feelings, to fully communicate risks and benefits, and to allow patients to make informed choices. This is not sufficiently emphasised in existing guidelines, and more emphasis needs to be placed in the future on respecting patients’ subjective wishes and developing a patient-centred comprehensive treatment plan.

Postoperative recurrence is a common phenomenon in desmoid tumor, occurring in 19%-77% of cases, mostly within 1.5-5 years after surgery (11, 21). The risk of recurrence is associated with tumor location and size, with larger tumors carrying a higher recurrence risk. A retrospective analysis by Cates and Stricker found no direct correlation between margin status and local recurrence, but close margins (<1mm) were associated with an increased risk of recurrence (22). Based on previous research and the cases presented, we believe that strict surgical resection is an essential treatment for desmoid tumor, and complete resection with negative margins can improve prognosis.

#### 3.5.2 Systemic therapy

While surgery was once considered the primary choice, its effectiveness is limited due to the high recurrence rate and potential for severe complications. In recent years, systemic therapy has gradually become an important part of the comprehensive treatment approach, not only for preventing recurrence but also as an initial treatment or adjuvant therapy after surgery. The main options include hormonal and non-steroidal anti-inflammatory drugs (NSAIDs), chemotherapy, and targeted therapy.

##### 3.5.2.1 Hormonal and non-steroidal anti-inflammatory drugs

Hormonal therapy includes selective estrogen receptor modulators (SERMs, such as tamoxifen, toremifene, and raloxifene) used alone or in combination with NSAIDs (e.g. meloxicam, indomethacin, and celecoxib). Bocale et al. reported on 168 patients with desmoid tumor treated with SERM monotherapy or in combination with other drugs, with an overall partial or complete response rate of 51% (23). NSAIDs have also been used to treat desmoid tumor, possibly by inhibiting cyclooxygenase-2 (COX-2) activity. Some studies have found that these drugs alone or in combination with SERMs can achieve a certain therapeutic effect, but there is currently insufficient evidence to demonstrate a significant difference in efficacy between the two approaches (24). Although hormonal therapy and anti-inflammatory drugs have been historically used, multiple studies have shown that their effectiveness is limited. An analysis found that the response rates of these two treatment methods were similar to those of the placebo arms and active surveillance. Therefore, those two modalities are not currently part of the main treatment options (17).

##### 3.5.2.2 Chemotherapy

Chemotherapy has been widely used in the treatment of desmoid tumors. Currently, methotrexate and vinblastine are among the commonly used chemotherapy regimens. A prospective study by Nishida et al. demonstrated that for desmoid tumor patients who progressed after prior treatment with meloxicam, combination chemotherapy with methotrexate (30mg/m^2^) and vinblastine (6mg/m^2^) administered every two weeks resulted in partial response in 40% of patients, with an additional 53.3% experiencing disease stabilization. The median progression-free survival exceeded 18 months, with good tolerability (25). Furthermore, the study found that the CTNNB1 mutation status did not significantly affect treatment outcomes. This is also consistent with the retrospective study findings of Nathenson et al, which explored the relationship between CTNNB1/APC mutation status and clinical benefits of systemic chemotherapy in patients with advanced abdominal wall desmoid tumors, and found that mutation subtypes did not affect chemotherapy efficacy (26). Additionally, Mir et al. conducted a retrospective study evaluating the long-term efficacy and toxicity profile of oral vinorelbine, a non-mutagenic cytotoxic drug, in treating advanced progressive desmoid tumors. The study included 90 patients with objective disease progression, and results showed that oral vinorelbine administered at 90mg per week achieved a 29% objective response rate and satisfactory progression-free survival benefit, with progression-free survival rates of 88.7% at 6 months and 77.5% at 12 months (27).

Using conventional doses of anthracycline-based chemotherapy is an important option and may lead to faster efficacy. A multicenter retrospective study by Garbay et al. revealed that the objective response rate of anthracycline-based chemotherapy regimens (54%) was significantly higher than non-anthracycline-based regimens (12%) (P=0.0011) (28). Gega et al. conducted a prospective study evaluating the efficacy and safety of the anthracycline drug doxorubicin (DOX) in combination with dacarbazine (DTIC) in treating unresectable desmoid tumors associated with FAP. The study included 7 such patients who received 4-5 cycles of DOX (20mg/m^2^, days 1-4) combined with DTIC (150mg/m^2^, days 1-4) chemotherapy. Results showed significant tumor reduction in all patients, with 3 achieving complete remission. The median progression-free survival was 74 months (32.5-107.5 months) (29). Additionally, in a retrospective study involving 12 patients with recurrent or refractory desmoid tumors, Constantinidou et al. reported the efficacy and safety of pegylated liposomal doxorubicin (PLD) monotherapy, administered at a dose of 50 mg/m^2^ every 4 weeks via intravenous infusion. Results showed that among the 11 evaluable patients, 4 (36%) achieved partial response, while 7 (64%) achieved disease stabilization, with a median progression-free survival of 14 months (30).

In conclusion, chemotherapy is an effective treatment option for desmoid tumors, but personalized treatment regimens should be selected based on individual patient characteristics and drug tolerability.

##### 3.5.2.3 Targeted therapy

With a deeper understanding of the molecular mechanisms underlying this disease, targeted therapies have also been applied in treatment. Several targeted drugs have been studied, including tyrosine kinase inhibitors (e.g. pazopanib and sorafenib) and γ-secretase inhibitors. Due to their convenience and limited long-term toxicity, these agents have been increasingly used as first-line treatments in recent years. In a non-comparative, randomized, open-label, multicenter, phase 2 study (DESMOPAZ study) evaluating the efficacy and safety of pazopanib and methotrexate-vinblastine combination chemotherapy for progressive desmoid tumors, 72 patients were enrolled and randomized to the pazopanib group (N=46) and the methotrexate-vinblastine group (N=24). In the 43 evaluable patients who received at least one cycle of pazopanib, the 6-month progression-free survival rate was as high as 83.7%, while it was 45.0% in the methotrexate-vinblastine group, and the pazopanib group had a lower incidence of adverse events. Overall, pazopanib demonstrated promising efficacy and safety in the treatment of desmoid tumors and could be considered as a first-line systemic therapy option (31). Sorafenib also demonstrates significant antitumor activity, prolonging progression-free survival in patients with advanced and refractory desmoid tumors. A randomized controlled study by Gounder et al. confirmed a remarkable 2-year progression-free survival rate of 81% in the sorafenib treatment group, far surpassing the placebo group’s 36%. Additionally, the objective response rate in the sorafenib treatment group was 33% (with a median duration of response of 9.6 months), higher than the placebo group’s 20% (with a median duration of response of 13.3 months) (32). However, a prospective nationwide clinical trial assessing active surveillance of efficacy, the GRAFITI trial, revealed that for patients with non-intraabdominal desmoid tumors, the 3-year progression-free survival rate was 58%, with an objective response rate of 28% (33). It is important to note that results from different clinical trials should be compared cautiously due to potential variations in patient populations and disease stages. Nonetheless, when considering treatment options, it is essential to weigh the risks of adverse reactions to sorafenib, which include common side effects such as rash, fatigue, hypertension, and diarrhea. Therefore, taking both efficacy and safety into account, sorafenib may not be the optimal choice for patients with desmoid tumors. Furthermore, in a randomized controlled trial involving 142 patients, nirogacestat (an investigational, oral, small-molecule, selective γ-secretase inhibitor) demonstrated significant benefits in treating adult patients with progressive desmoid tumors (34).

While systemic treatments can control the progression of desmoid tumor to a certain extent, they are often associated with adverse effects and drug resistance issues. Therefore, despite their efficacy, systemic therapies still have significant limitations and require careful consideration of the risks and benefits for individual patients.

#### 3.5.3 Radiation therapy

Radiation therapy can be used alone for unresectable tumors or patients who cannot tolerate surgery, but the use of postoperative radiation therapy remains controversial. Due to the unique location of the tumors, radiation therapy increases the risk of complications in other intra-abdominal organs, and radiation itself is a carcinogenic factor. Therefore, radiation therapy is not recommended for the treatment of intra-abdominal desmoid tumor (35, 36).

## 4 Conclusion

Desmoid tumor is a rare but clinically challenging tumor, particularly when it occurs in the mesentery. The clinical presentation lacks specificity, making preoperative diagnosis difficult. For patients with rapidly progressing tumors, prompt surgical resection is currently recognized as the primary treatment approach. However, due to the infiltrative growth pattern and high recurrence rate of these tumors, close postoperative follow-up and subsequent treatment is essential. Therefore, further large-scale research is needed to optimize the diagnostic and treatment protocols for desmoid tumor, ultimately enhancing patient survival and quality of life.

## Data availability statement

The original contributions presented in the study are included in the article/supplementary material. Further inquiries can be directed to the corresponding author.

## Ethics statement

Written informed consent was obtained from the individual(s) for the publication of any potentially identifiable images or data included in this article.

## Author contributions

C-YL: Conceptualization, Writing – original draft. Y-PG: Data curation, Investigation, Writing – review & editing. M-HJ: Data curation, Writing – review & editing. YZ: Data curation, Writing – review & editing. J-YX: Supervision, Writing – review & editing, Project administration.
